# Feasibility and acceptability of an adapted environmental enrichment intervention for endometriosis: A pilot study

**DOI:** 10.3389/fgwh.2022.1058559

**Published:** 2023-01-04

**Authors:** Cristina I. Nieves-Vázquez, Amanda C. Detrés-Marquéz, Annelyn Torres-Reverón, Caroline B. Appleyard, Astrid P. Llorens-De Jesús, Ivana N. Resto, Verónica López-Rodríguez, Paola M. Ramos-Echevarría, Eida M. Castro, Idhaliz Flores

**Affiliations:** ^1^ Public Health Program, Ponce Health Sciences University, Ponce, Puerto Rico; ^2^ School of Medicine, Ponce Health Sciences University, Ponce, Puerto Rico; ^3^ Department of Basic Sciences, Ponce Health Science University, Ponce, Puerto Rico; ^4^ Sur180 Therapeutics, LLC, McAllen, TX, United States; ^5^ School of Behavioral and Brain Sciences, Ponce Health Sciences University, Ponce, Puerto Rico; ^6^ Department of Obstetrics and Gynecology, Ponce Health Sciences University, Ponce, Puerto Rico

**Keywords:** endometriosis, environmental enrichment, feasibility, acceptability, randomized clinical trial, psychosocial

## Abstract

**Introduction:**

We have previously shown that Environmental Enrichment (EE)-consisting of social support, novelty, and open spaces—decreased disease progression and anxiety in a rat model of endometriosis. We developed a novel EE intervention to be tested in a pilot randomized clinical trial (RCT) in patients with endometriosis, a painful, stressful disease.

**Objective:**

To translate and evaluate the feasibility and acceptability of an adapted EE intervention as an adjuvant to standard-of-care for endometriosis patients.

**Methods:**

Feasibility was assessed through recruitment, enrollment, and adherence rates. Acceptability was evaluated through a post-intervention survey and focus group discussion 3-months after the end of the intervention.

**Results:**

Of the 103 subjects recruited, 64 were randomized to the intervention group and 39 to the control group. At the start of the intervention, the study groups consisted of 29 (intervention) and 27 (control) subjects. Enrollment rates were 45.3% and 69.2%, and adherence rates were 41.4% and 100% for the intervention and control groups, respectively. Delays resulting from natural events (earthquakes, the COVID-19 pandemic) impacted enrollment and adherence rates. The most common reasons for missing an intervention were period pain (39.1%) and work-study (34.8%). There was high acceptability (>80%) of the intervention's logistics. The majority (82.4%) of subjects would continue participating in support groups regularly, and 95.7% would recommend the intervention to other patients.

**Conclusions:**

We showed that EE could be translated into an acceptable integrative multi-modal therapy perceived as valuable among participants who completed the intervention. High attrition/low adherence indicates that additional refinements would be needed to improve feasibility. Acceptability data indicate that EE has the potential to be integrated into the clinical management of patients with endometriosis and other inflammatory, painful disorders. Studies are ongoing to assess the efficacy of EE in improving pain symptoms, mental health, and quality of life (QoL).

## Introduction

Environmental enrichment (EE) is defined as a “combination of inanimate and social stimulation” involving a socially integrated lifestyle, physical and cognitive stimulation, and larger spaces (1–4). In animal models, EE improves cognition and memory, symptomatology of stroke, Alzheimer's disease (AD), Huntington's disease, chronic stress, visceral/inflammatory pain, depression, and anxiety (5–17). A translated EE intervention was recently shown to increase physical, social, and cognitive levels in acute stroke patients and to decrease adverse events compared to the standard of care counterparts (18–21). A recent study associated levels of EE exposure with depressive symptoms through an EE indicator that measured cognitive, social, and physical activity and could differentiate participants with major depression from control subjects (22). Still, very few studies of multi-modal interventions involving features of EE have been conducted in humans, and none in pain or inflammatory disorders.

The mechanisms by which EE provides its benefits are hypothesized to be at multiple levels, with strong evidence showing modulation of immune pathways to produce anti-inflammatory effects (23, 24), as well as at the central level through increased expression of brain-derived neurotrophic factor (BDNF) and decreased levels of corticotropin-releasing factor receptor type 1 (CRFR1) in amygdala (6, 16). In addition, EE causes “eustress”, the type of stressor that provides hope and a feeling of fulfillment (e.g., a new opportunity linked to a positive outcome) (25, 26). Eustress positively affects the quality of life (QoL), psychological coping, and mental health, increased level of resiliency to challenges. Independently, features of EE—large spaces for therapy, use of multi-sensorial equipment, active engagement in novel activities, and enhanced social interactions—have been used in the clinic (27, 28). However, despite ample evidence of EE effectiveness in animal models and the encouraging results in patients with stroke, there is still an unmet need to adapt the EE paradigm to the human scenario and generate outcomes on its clinical efficacy for stress-related and inflammatory disorders.

The painful symptoms of endometriosis—severe dysmenorrhea, dyspareunia, and chronic pelvic pain—lead to high stress due to their negative impact on psychological wellbeing, social functioning, and QoL (29–31). Stress alters gut and uterus motility, exacerbates inflammatory parameters, and is known to increase visceral pain perception (10, 32–35). We and others have shown that women with endometriosis or dysmenorrhea have low levels of cortisol compared to controls (36–38). Moreover, cortisol levels were negatively correlated with symptoms (dyspareunia, infertility) in patients who also showed elevated anxiety state scores (38). Therefore, it has been suggested that stress management could improve endometriosis symptomatology and general wellbeing of endometriosis patients. Our group conducted animal studies showing for the first time the contribution of stress to the development and progression of endometriosis (34, 35, 39). Stress increased vesicle size and number, colonic inflammation and motility, inflammatory cell infiltration into vesicles, expression of nerve growth factor (NGF) and its receptors in uterus. It also deregulated hypothalamic-pituitary-adrenal (HPA) axis responses in rats with endometriosis. Importantly, we showed that the ability to control the stress reversed these parameters resulting in smaller vesicles and decreased cellular infiltration and inflammation (40).

Together, our research using the rat model of endometriosis suggest that while stress contributes to disease development and severity in the rat model through mechanisms involving inflammation, nerve growth, and HPA axis deregulation, stress controllability improves disease progression, thus offering possibilities for therapeutic interventions based on this paradigm (41). Given these promising results, we hypothesized that EE would stabilize HPA axis activity and reduce lesion development in a rat model of endometriosis. We found that in rats with experimental endometriosis exposed to EE, the number and size of developed endometriotic vesicles were significantly decreased compared to those under control conditions (42). In addition, rats in the EE group spent more time in the center of an open field box, suggesting decreased unconditioned anxiety behaviors. These results strongly suggest that the combination of elements in EE could also have beneficial effects by reducing stress while improving mental health and disease severity in the clinical scenario.

To address this question, we proposed to adapt and test a multi-level structured program based on the EE paradigm for endometriosis patients. Using an integrative, patient-centered methodology, we developed an EE intervention program consisting of six modules, with activities mimicking and integrating the three hallmarks of EE: social support, novelty, and open spaces. Here, we describe the acceptability and feasibility of a novel, translated EE intervention to be used as an adjuvant to standard care in women with endometriosis, an inflammatory, painful disease associated with chronic stress. Future analysis of efficacy data collected from this randomized clinical trial (RCT) will assess whether EE promotes stress management, immune modulation, pain relief, and improvements in mental health and QoL for women with endometriosis.

## Methods

### Translation of the EE intervention

A team composed of investigators with expertise in psychology, physiology, neuroscience, integrative mind-body, gynecology, translational research, and stress management, met for a year to translate the environmental enrichment (EE) paradigm that was proven efficacious in the rat model to the clinical setting. A pilot RCT was designed to test (1) acceptability and feasibility of the intervention and (2) EE's efficacy for reducing painful symptoms and improving QoL. The adaptation followed the ORBIT Model for developing behavioral interventions for chronic diseases (43). This model guides the early pre-efficacy stages of behavioral intervention development using a four-phase approach (F1: design, F2: preliminary efficacy, F3: efficacy, F4: effectiveness. Guided by the model, we translated a basic neurobehavioral finding (*EE reduces the growth of lesions in rats*) into a clinical question (*will an EE intervention effectively reduce pain symptoms/inflammation and increase QoL in endometriosis?).* First, we conducted a systematic literature review to select the approaches for the adapted EE intervention, using the PRISMA-P 2015 protocol and the PRISMA evaluation checklist (44, 45). Through this process, we identified treatment components to reflect the three hallmarks of the EE paradigm (social support, novelty, open spaces) that were tested in RCTs for pain, inflammation, or chronic disorders: *yoga, yogic breathing, mindfulness, aromatherapy, art therapy, music and drama therapy, support groups* and *the outdoors* (46–59). Decisions on the interventions to be included in the final program were based on the following factors: (1) evidence that the activity was effective for improving symptomatology and QoL, even if had not been tested in endometriosis, (2) local availability of certified coaches, (3) time limitations (up to 3 h), and (4) involving gentle physical activity. A team composed of the PI, Research Coordinator, and Wellness expert scouted potential venues to identify those with open spaces overlooking natural areas and located within an hour's drive from Ponce. The Patient Advisory Committee (PAC), composed of five members of the *Fundación Puertorriqueña de Pacientes con Endometriosis* (ENDOPR), a patient support foundation in Puerto Rico, evaluated the proposed activities (no concerns), venues (all approved), preference of date/time (Thursday PM or Saturday AM), frequency (every two weeks) and duration (3 h) of the EE modules.

### Recruitment

After IRB approval (Protocol #1901004205R003), we conducted a recruitment campaign using social media (Facebook, Instagram, Twitter) of ENDOPR. Recruitment for Session I took place from September 2019 to December 2021. We had to delay the start of the RCT due to a series of earthquakes (January–February 2020) and the COVID-19 pandemic (March 2020). A second recruitment campaign was conducted from February to June 2021. Recruitment for Session II took place from August 2021 to February 2022. Patients interested in participating were screened for inclusion/exclusion criteria and consented prior to randomization into the intervention or control groups.

### Inclusion and exclusion criteria

Participants were women with a surgical diagnosis of endometriosis, 18–50 years old, symptomatic (currently experiencing pelvic pain despite treatment), who could commit the time to participate in EE intervention for three months. We excluded patients who were pregnant or planning to become pregnant during the study period; post-menopausal; with documented impairments that would interfere with participation or consent; under psychiatry treatment; using steroid medications. We also excluded those affected by other confounding conditions, including pain syndromes (e.g., fibromyalgia, chronic fatigue syndrome, arthritis).

### Study design of the pilot RCT

To evaluate the feasibility, acceptability, and efficacy of a translated EE intervention for endometriosis patients, we conducted a RCT of parallel design with an attention control group (standard of care plus educational webinar) and an intervention group (EE intervention) from August 2021 to July 2022. Participants completed surveys to assess: (1) clinical history (Endometriosis Phenome Project Clinical Questionnaire -EPhect-Q) (60), (2) QoL (Endometriosis Health Profile-30, EHP-30), (3) pelvic pain (Brief Pain Inventory, BPI), (4) perceived stress (Perceived Stress Scale-14, PSS-14), (5) co-morbidities, and (6) mental health symptomatology (General Anxiety Disorder-7, GAD7, and Patient Health Questionnaire-8, PHQ-8). Biospecimens (saliva for cortisol, serum for inflammatory cytokines) were collected at baseline, end of the intervention (6 modules), and three months after the end of the intervention. We conducted group assignments by computer-generated permuted block randomization. Subjects randomized to the intervention condition received the EE intervention as an adjuvant to standard gynecological care consisting of hormonal, analgesic, or surgical treatment. Participants randomized to the control condition received standard gynecological care and were invited to participate in an online seminar about endometriosis (“Becoming an Endometriosis Patient Expert” delivered by the PI). For ethical reasons, patients allocated to the controlled group were offered the EE intervention after completing the follow-up assessments. We collected samples (blood, saliva) and study surveys from subjects in the control group during home visits. For this group, we also assessed feasibility. Primary and secondary outcomes of the EE intervention—pain and QoL improvement, mental health (depression and anxiety), and stress improvements, as well as correlations with cortisol changes and inflammatory markers—will be assessed in ongoing efficacy studies.

### Study subject demographics

Sociodemographic and clinical data obtained from subjects in the intervention and control groups through the Endometriosis Phenome Project (EPHect) questionnaire were analyzed using descriptive statistics (means, %, frequency, proportions).

### Feasibility measures

We evaluated the feasibility of the EE intervention at three levels: recruitment, enrollment, and adherence to treatment. For all metrics, we defined feasibility as Definitely, Possibly, or Not feasible (>70%, 50%–69%, or <50% of eligible participants recruited, respectively). Recruitment feasibility was the ratio of the number of subjects who met the inclusion criteria and the number of eligible subjects. Enrollment feasibility is the proportion of eligible patients who agreed to participate in the study. Adherence to treatment and retention were monitored by keeping a record of dropouts, loss to follow-up, attendance to sessions, completion of surveys, and completion of the intervention (six modules). The reasons for missing sessions or dropping out were recorded.

### Acceptability measures

We assessed participants' satisfaction three months after the end of the intervention with a survey and during focus group meetings. Participants assessed the EE intervention logistics and delivery process, including the day/time, location, and frequency of meetings.

### Qualitative data analysis

During the post-intervention focus group meetings, group facilitators asked subjects to evaluate their perceived value of the EE intervention. All audio transcripts were transcribed and verified by advanced psychology students. A thematic analysis was conducted to interpret the transcripts from the follow-up sessions and the open-ended questions of the evaluation survey. This technique allows the researcher to identify, analyze and report the themes that emerge from the recollected data. To achieve this, the Braun & Clark (2021) (61) six-phase conceptual framework was used. From this approach, the thematic analysis process focuses on the relevance and importance of the identified themes and how they relate to the objective of the study. In this study, the relevant themes were associated with the feedback provided by the participants based on their experience during the environmental enrichment interventions and the support groups. Two advanced psychology students transcribed verbatim and checked for accuracy to minimize bias. Then, three research team members read the transcripts and independently identified initial categories. Finally, the team discussed and identified the final categories. After being 100% in agreement, the final themes and sub-categories were selected and NVivo 12 software was used to systematically organize the data using specific verbatims.

## Results

### Development of the EE intervention modules

**Figure 1:**
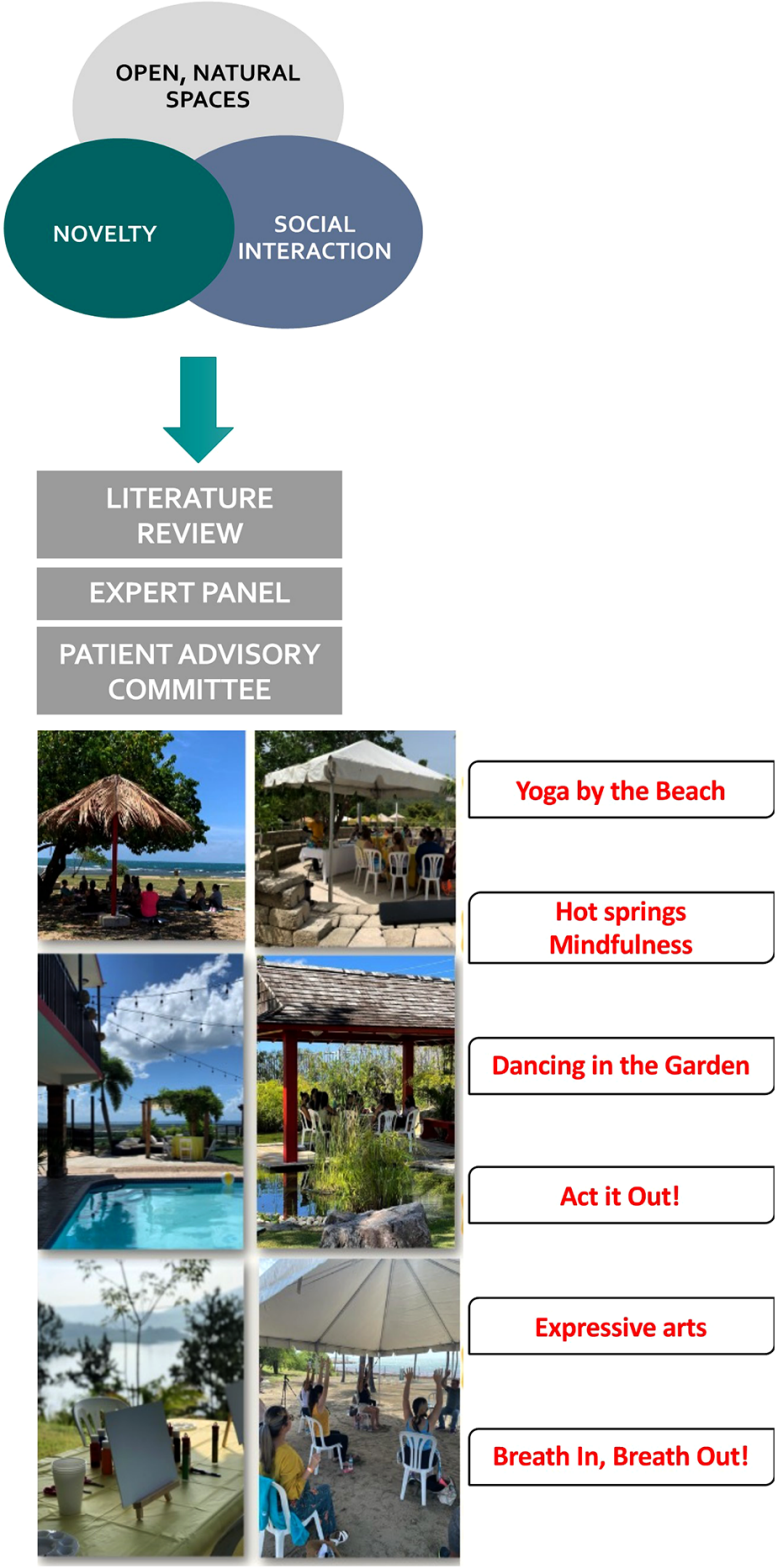
Translation of the EE paradigm to humans. A multidisciplinary team of experts, with input from a patient advisory committee, translated an enriched environment intervention based on the EE paradigm consisting of social support, novelty, and open spaces to be tested in a pilot psychosocial trial. EE interventions took place in open space venues (e.g., beach, lake, garden, hot springs, countryside) and consisted of a support group meeting (1 ½ h) and a novel stress-management activity (1 h). The interventions were conducted every other Saturday morning for a period of 6 months.

We developed six EE modules, integrating knowledge from a literature review, expert opinion, and input from the PAC. Each module reflected the three hallmarks of the EE paradigm: social support, novelty, and open spaces. The PAC evaluated and approved the proposed activities, venues, date/time, frequency, and duration of the EE modules. They considered that the intervention addressed the needs, concerns, and expectations regarding complementary approaches for endometriosis symptom management. The final intervention consisted of six modules administered every other Saturday morning that included: a support group meeting (1 ½ h) and a novel stress-management activity (1 h) that took place in open space venues: beach, lake, garden, hot springs, countryside (Figure 1). For the final program, the team selected the following activities: *yoga, yogic breathing, mindfulness with aromatherapy, art therapy, drama therapy, and dance therapy*.

### Recruitment, enrollment, and retention

**Figure 2:**
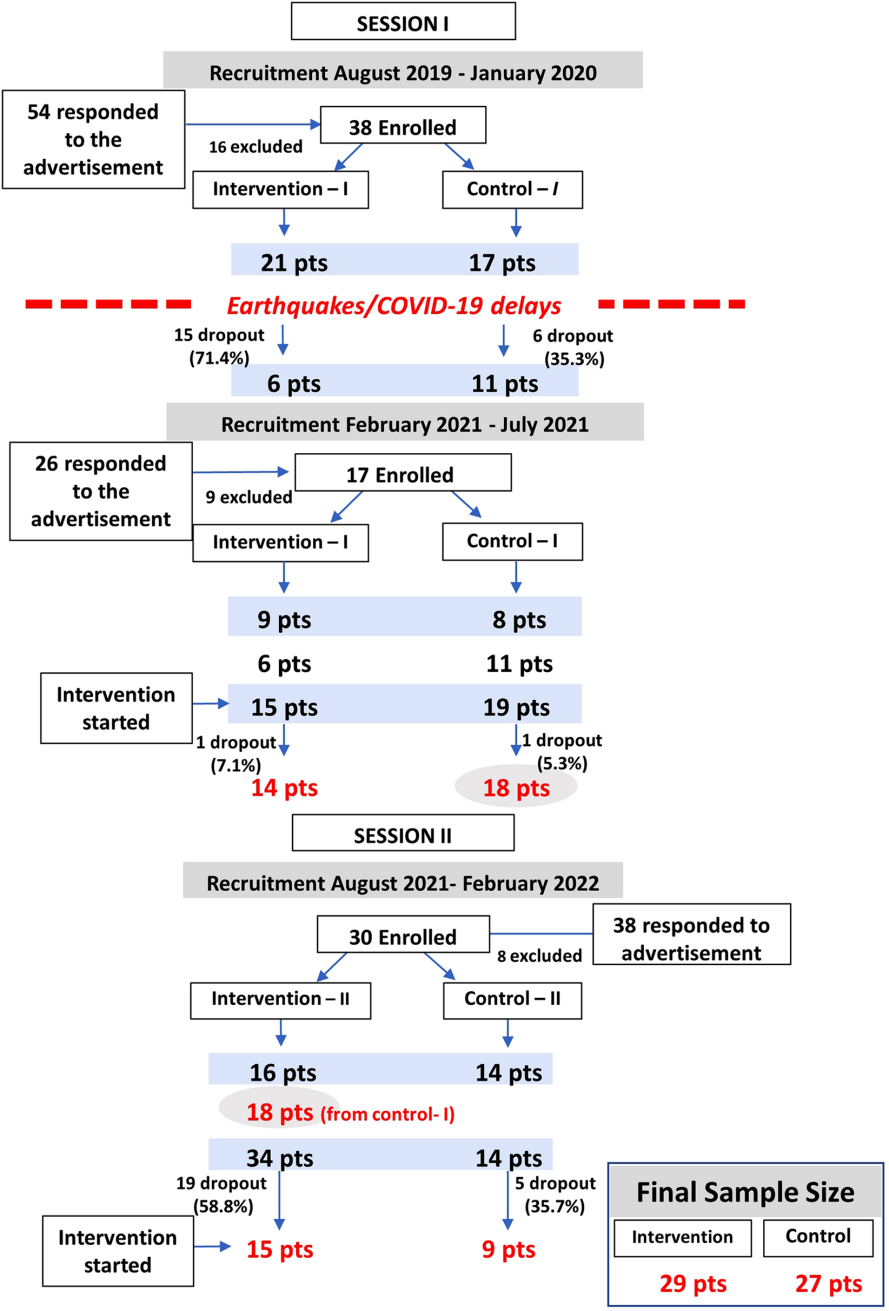
Enrollment diagram. The recruitment, enrollment, and retention of the EE pilot RCT are shown. Recruitment was conducted *via* social media of a patient support foundation. There were two recruitment efforts for Session I which took place from August to October 2021, and one for Session II which took place February to April 2022. A total of 118 individuals were screened and 103 were randomized to the EE vs. control groups. Final enrollment was *n* = 29 (EE) and *n* = 27 (control). Dropout rates were 54.7% for the EE group and 30.8% for the control group.

The RCT was conducted in two sessions over 8 months (August 2021–April 2022). Overall, of 118 individuals responding to the study advertisements, 103 (85 + 18 waitlist controls) were randomized into the intervention (*n* = 64) and control (*n* = 39) groups for an exclusion rate of 28.0%. Dropouts (including those excused, no-shows, and those not completing the consent form) were 35 (54.7%) in the intervention group and 12 (30.8%) in the control group. The final enrollment was 29 subjects in the intervention and 27 in the control groups, and adherence rates (subjects who completed the six interventions) were 41.4% and 100%, respectively (Figure 2, Table 1).

**Table 1:** Recruitment, enrollment, and retention of the EE intervention.

Study group	Total randomized	Drop-outs	Total enrolled	Enrollment rate	Dropout rate	Completed intervention	Adherence rate
Intervention	64	35	29	45.3%	54.7%	12	41.4%
Control	39	12	27	69.2%	30.8%	27	100.0%
Total	103	48	56	55.6%	44.4%	39	

The recruitment, enrollment and retention of study subjects in both the Intervention and Control groups are shown.

For the first session, a total of 54 individuals responded to our initial advertisement from August 2019 to December 2019; 38 met the inclusion criteria (70.4%) and were randomized into the intervention (*n* = 21) and control (*n* = 17) groups. The intervention was planned to start on January 11th, 2020. Unfortunately, Puerto Rico suffered a series of earthquakes starting on January 7th that forced the postponing of the intervention. On March 2020, Puerto Rico entered a lockdown mandated by the government due to COVID-19, forcing a second postponing of the intervention. These delays led to dropouts (*n* = 21, 55.3%) for reasons including pregnancy, relocation, and work/study issues. A second recruitment effort was conducted from February 2021 to July 2021, in which 26 individuals contacted our program; 17 met inclusion criteria (65.4%) and were randomized to the intervention (*n* = 9) and control (*n* = 8) groups. There were two other dropouts after the intervention started. Overall, for Session I, 80 individuals contacted our program, and 55 (68.8%) were randomized to the intervention (*n* = 30) and control (*n* = 25) groups. Dropout rates for Session I were 53.3% (*n* = 16) and 28.0% (*n* = 7) in the intervention and control groups, respectively. The final study groups were 14 subjects in the intervention group and 18 in the control group for Session I (August to October 2021).

For Session II, a third recruitment session took place from August 2021 to February 2022. Of the 38 individuals responding to the study advertisements, 30 (79.2%) met the inclusion criteria and were randomized to the intervention (*n* = 16) and control (*n* = 14) groups. The attention control group from Session I (*n* = 18) was invited to participate in the Session II intervention, for a total of 34 subjects randomized to the intervention group. Dropout rates for Session II were 55.9% (*n* = 19) in the intervention group and 35.7% (*n* = 5) in the control group for reasons including work/study, distance to intervention locations, or lost to follow-up. No subjects dropped out during Session II. The final study groups were 15 subjects in the intervention group and 9 in the control group for Session II (February to April 2022).

### Study subject characteristics

The clinic-demographic data from subjects in the study groups are shown in Table 2. No significant differences were observed between the study groups, except for the proportion of subjects with post-graduate education. Baseline mean levels of current pelvic pain measured by the Brief Pain Inventory (BPI) were 8.0 (SD ± 2.2) and 6.4 (SD ± 3.3) for the intervention and control groups, which was not significantly different. Baseline perceived stress levels measured by the Perceived Stress Scale 14 (PSS-14) were 32.5 (SD ± 5.7) and 28.0 (SD ± 4.4) for the intervention and control groups, which was not significantly different.

**Table 2:** Clinic-demographic characteristics of the study population.

Characteristic	Intervention group (*n* = 29)	Control group (*n* = 27)
**Mean age (yrs)**	32.7 (range: 21–45)	34.6 (range: 18–49)
**Median BMI**	24.7 (SD ± 6.9)	24.8 (SD ± 6.5)
**Private insurance**	62%	73%
**Committed relationship**	45%	46%
**Currently studying**	37.9%	34.6%
**Currently working**	89.7%	80.8%
**Education (post-graduate)**	32%	68%*
**Regular Cycle**	55.2%	69.2%
**Ever pregnant**	21%	27%
**Age symptom onset (yrs)**	18.2	17.9
**Age at diagnosis (yrs)**	24.2	26.0
**Pelvic pain**	100%	92.3%
**Dyspareunia**	75.9%	73.1%

The self-reported demographic and clinical characteristics of the study participants is summarized.

BMI, body mass index; Yrs, years; SD, standard deviation.

* *p* = 0.01.

### Feasibility measures

Table 3 summarizes the metrics of feasibility: recruitment success (how many qualified and were randomized), enrollment rates (how many started the intervention), and adherence rates (how many completed the intervention), for each of the study groups.

**Table 3:** Feasibility results.

Measure/Study group	Calculation (×100)	%	Feasibility
Recruitment rate overall	85 subjects randomized/118 subjects screened	72.0	Feasible
Enrollment rate intervention	29 subjects enrolled/64^a^ subjects randomized	45.3	Not feasible
Enrollment rate control	27 subjects enrolled/39 subjects randomized	69.2	Possibly Feasible
Adherence rate intervention group	12 participants completed the assigned treatment/29 of participants allocated to treatment	41.3	Not feasible
Adherence rate control group	27 participants completed the assigned treatment/27 of participants allocated to treatment	100	Feasible
Retention rate intervention group	12 subjects at the end of the study/64 of subjects randomized	18.8	Not feasible
Retention rate control group	27 subjects at the end of the study/39 of subjects randomized	69.2	Possibly feasible

Summary of the feasibility data, including recruitment, enrollment, adherence and retention rates.

^a^ Includes the attention control group.

### Acceptability measures

We evaluated participation rate, completion rates of the study surveys per intervention (baseline, mid-term, end, 3-month follow-up), and acceptability of the intervention using a post-intervention evaluation survey and focus group.

#### Participation rate

**Figure 3:**
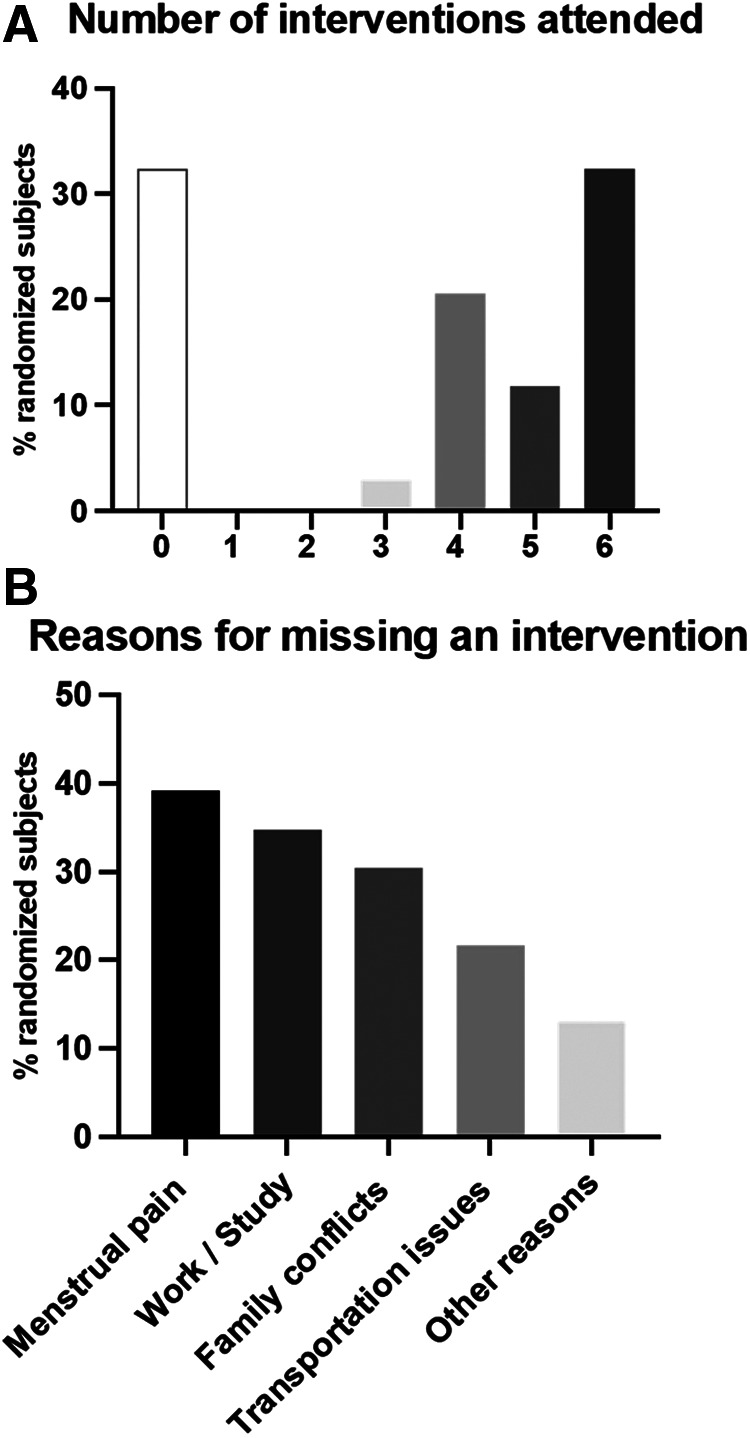
Participation rate of study subjects in the EE intervention. Full dose and half dose are shown in panel **A**. Reasons for missing an intervention were documented *via* an online survey are shown in panel **B**.

A total of 12 subjects in the EE group completed all six interventions (full dose) (41.4%), and 17 participants completed at least three interventions (half dose) (58.6%) (Figure 3A). The most common reasons for missing an intervention were period pain (39.1%; *n* = 9), work-study (34.8%, *n* = 8), and family commitment conflicts (30.4%, *n* = 7) (Figure 3B).

#### Survey completion rates

Table 4 summarizes the survey completion rate by study group. In general, we observed high response rates for all surveys (>80%) except for the 3-month follow-up after the end of the intervention time point.

**Table 4:** Survey completion rates.

Survey	Intervention (*n* = 29)	Control (*n* = 27)
**EPhect MQ**	29 (100%)	27 (100%)
**BPI**		
Baseline	27 (93.0%)	22 (84.6%)
End	27 (93.0%)	25 (93.0%)
3-mo. follow-up	16 (55.1%)	11 (40.7%)
**PSS-14**		
Baseline	29 (100%)	25 (96.0%)
End	26 (89.7%)	23 (85.0%)
3-mo. follow-up	17 (59.0%)	11 (40.7%)
**EHP-30**		
Baseline	29 (100%)	20 (76.9%)
End	26 (89.7%)	26 (96.3%)
3-mo. follow-up	17 (58.6%)	14 (51.9%)
**Clinical history**	28 (97.0%)	25 (93.0%)
**GAD-7**		
Baseline	29 (100%)	27 (100%)
End	29 (100%)	27 (100%)
3-mo. follow-up	19 (65.5%)	N/A
**PHQ-8**		
Baseline	29 (100%)	27 (100%)
End	29 (100%)	27 (100%)
3-mo. follow-up	19 (65.5%)	N/A

Summary of completion rates of the study surveys. EPhect-MQ, endometriosis phenome project minimal questionnaire; BPI, brief pain inventory; PSS-14, perceived stress scale 14; EHP-30, endometriosis health profile 30; GAD-7, general anxiety disorder 7; PHQ8, patient health questionnaire depression scale 8; mo. = month.

#### Intervention evaluation survey

**Figure 4:**
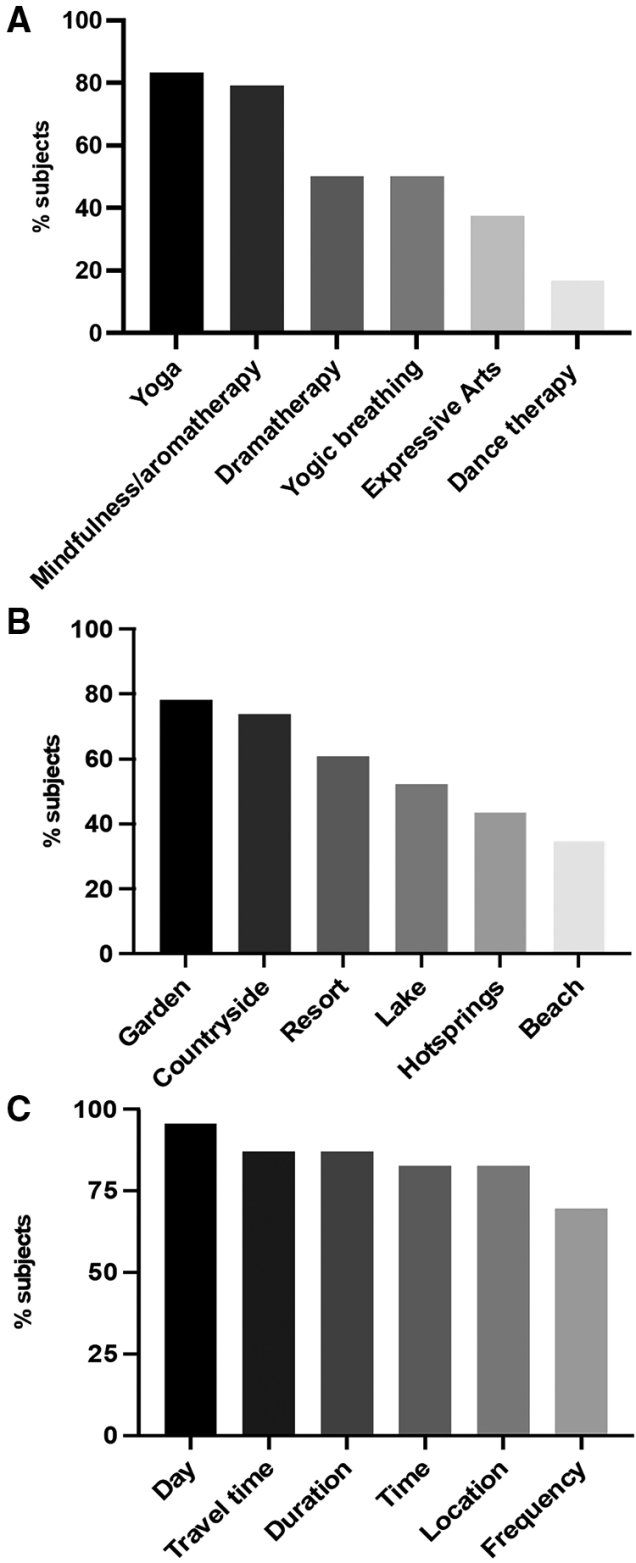
Intervention evaluation survey results are shown. Favorite modules are shown in panel **A**. Favorite venues are shown in panel **B**. Acceptability of logistics are shown in panel **C**.

Subjects allocated to the EE group (*n* = 29) were sent an electronic link to a post-intervention survey. The favorite module was yoga (83.3%, *n* = 20) (Figure 4A); the favorite location was the Japanese garden (78.3%, *n* = 18) (Figure 4B). Figure 4C shows the acceptability of the study logistics ranging from 95.7% (*n* = 22) for the intervention day (Saturday) to 69.6% (*n* = 16) for the frequency (every two weeks). The support group component of the intervention was evaluated as *Excellent* by 90.5% (*n* = 19/21). When asked if they would recommend the EE intervention to other patients 95.7% (*n* = 22/23) of them said *Yes*. When asked about desire to have future participation in support groups with other women with endometriosis 82.4% (*n* = 28/34) said they would participate regularly (this included subjects who were randomized to the intervention but did not participate). Salient verbatims of the answers to an open question on the survey (Any comments on the support group experience?) are included in Table 5.

**Table 5:** Feasibility of the EE intervention: analysis of open-ended question answers in the final evaluation survey.

Verbatims
*“Considero los grupos de apoyo una parte esencial en este proceso. Sin duda, una de mis actividades favoritas. Me ayudó mucho. Empatía al 100%.”* [*“I consider support groups an essential part of this process. Without a doubt, [it was] one of my favorite activities. It helped me a lot. [There was] 100% empathy.”*]
*“Fue muy sanadora la experiencia en los grupos de apoyo. Ya no me siento sola y, además, logré conectar conmigo misma y aceptarme tal y como soy. Mis niveles de frustración han disminuido y he aprendido a disfrutar mi tiempo libre y la naturaleza.”* [*“The experience in the support groups was very healing. I no longer feel alone; I also managed to connect with myself and accept myself as I am. My frustration levels have decreased, and I have learned to enjoy my free time and nature.”*]
“Las actividades del grupo de apoyo fueron excelentes.” [*“Support group activities were excellent.”*]
“Eso fue muy bueno para mí. Jamás pensé ser tan libre, poder hablar con tanta confianza y que fuera así de escuchada. ¡Estoy tan y tan agradecida!” [*“This was very good for me. I never thought to be so free, to speak with such confidence and be so listened to. I am so, so thankful!”*]
*“Mi parte favorita. Necesaria. Indispensable. De mucha ayuda. [Hubo] Empatía al 100%.”* [*“My favorite part. Necessary. Indispensable. Of much help. [There was] 100% empathy.”*]
*“Me parecieron buenas para abrir un espacio seguro para que podamos compartir nuestras experiencias.”* [*“I thought they were good for opening up a safe space for us to share our experiences.”*]
*“Son excelentes. Su trato es especial.”* [“*They are excellent. They treated us in a special way.”*]

Selected comments regarding the support groups in the post-intervention evaluation survey.

#### Exit interview evaluation focus groups

**Figure 5:**
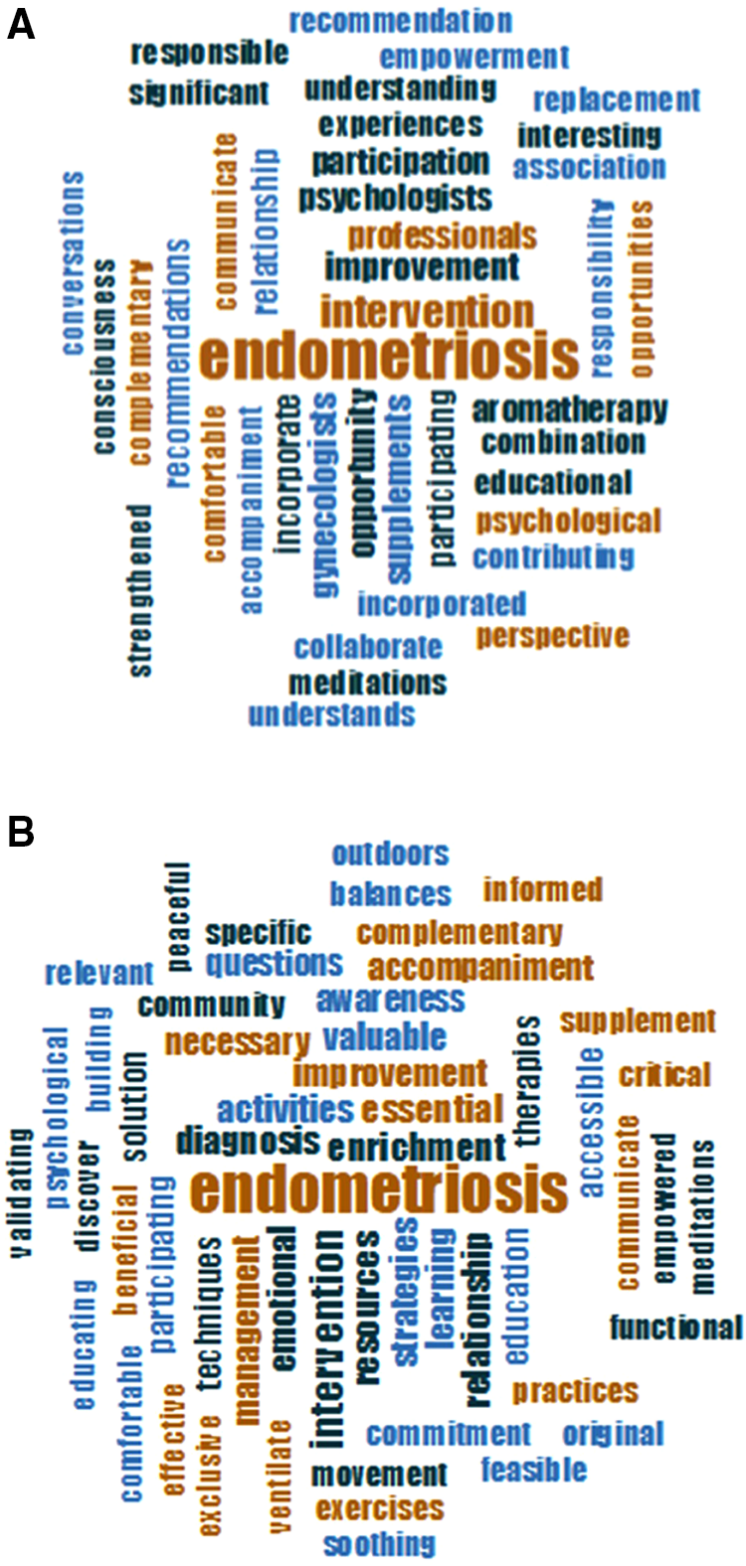
Word clouds representing the most common words used to describe the subjects’ experience during the EE intervention. Transcripts from support group discussion that took place 3 months after the end of the intervention were analyzed using NVivo. Perceived benefits of the EE intervention are shown in panel **A**. Perceived value of the EE intervention for the management of endometriosis symptoms is shown in panel **B**.

During the 3-month follow-up session, 12 patients from the intervention group only participated in focus group discussions to gather qualitative data regarding their acceptability and perceived value of the EE intervention. Qualitative analysis of the exit focus group transcriptions using NVivo-12 is shown in Figure 5 and Table 6. Perceived relative importance is reflected in the font size. The most beneficial aspect of the intervention was the perceived improvement of endometriosis symptoms. The themes identified were *accompaniment*, *consciousness*, *responsibility*, and *understanding*. “*Not feeling alone*” was one of the aspects they valued the most from the *accompaniment* theme. Effects of EE on the management of their condition were categorized in these themes: *validating*, *effective*, *soothing*, *critical*, *accessible*, *functional*, and *valuable*, which evidence a high acceptability of the interventions.

**Table 6:** Feasibility of the EE intervention: analysis of 3-month after the end of the intervention group discussion transcripts.

Theme	Verbatim
Companionship	“*…and listening to my companions because this was a luxury.”* “*Knowing that those resources are there if you need help or if I feel alone, I know who I can talk to that will understand my emotional needs… that honestly gives a lot of meaning to this.”* “*It was a space where we could express ourselves as we wanted with people with whom I could identify.”* “*So, for me, the most important thing was the accompaniment, the feeling that there are people who can understand me and that professionals are making an effort so that other professionals are educated so that we are well.”*
Self-compassion	“*It is not only the physical weakening but the emotional, self-esteem and then that we can generate self-compassion also with our bodies. Because we are enough despite this condition…”* “*But the way I talk to myself when I'm in pain has changed a lot more because I don't feel the pressure to be functional all the time.”*
Safe space	*“It was a learning space. It was a safe space and, above all, the most important thing was that it was free of judgment.”* “*It's a safe space because we're all on the same page because of the condition. It allowed us to ventilate, just to be there. Because sometimes it's not even talking, only being there, I think it's super necessary.”*
Self-efficacy	*"Being able to say to the support group ‘this is what I do when I feel bad’ that maybe someone else didn't know and being able to apply that same knowledge in one's home has been great for me.”* “*Before participating in the intervention, I came to the beach and loved it. Still, I was not aware of my presence, of my body. For me, this was a very complementary practice and perfect combination.”* “*It gave me the sense of being able to take responsibility and educate, of not staying silent, of guiding others.”*
Therapeutic value	*"This environmental enrichment helped me heal, be more at peace with myself, understand myself better, and be more empathetic in the process.”* “*I feel like it was a step that gave me a kind of “jamaqueón” (It shook me)*. “*Mind-body: that's essential.”*
Research	*"In addition to the support group that I loved, it's also knowing that I'm not the only one and sharing experiences and information. I, at least personally, also feel good about contributing to the research.”* “*This is something that's going to help for the greater good.”*

Main themes and representative verbatims from participants during post-intervention evaluation discussion. Main themes were identified by analysis of discussion transcripts using NVivo.

### RCT safety

During the two sessions of the EE intervention there were no severe adverse events (SAE), and no patient had to stop treatment due to an adverse event (AE). Only one AE was reported during the aromatherapy intervention: a mild and transient irritation to an essential oil.

## Discussion

Here we report the acceptability and feasibility results of the first clinical study of an enriched environment intervention providing social support, novelty, and open spaces in patients with endometriosis. Based on our previous findings that EE can effectively reduce disease progression and anxiety behaviors in a rat model (42), our multidisciplinary team developed an integrative multi-modal intervention based on this psychosocial paradigm. Through this pilot RCT, we demonstrated that the adapted EE intervention is acceptable as an adjuvant to standard care for endometriosis. However, feasibility results, shown by enrollment and adherence rates, indicate that additional refinement would be needed to facilitate its implementation. Data from this study and follow-up studies on efficacy involving a larger sample size will inform the implementation of mind-body integrative medicine strategies in the standard of care for this painful and challenging condition.

Our team was the first to experimentally demonstrate that stress increases the size of endometriosis lesions and worsens inflammation and anxiety (34, 35). We also showed that the “controllability” of stress can influence endometriosis pathophysiology, offering the possibility of using stress management and coping techniques as complementary approaches in patients with this condition (40). Other investigators have also shown the beneficial effects of stress management and EE on other rodent models of endometriosis (62). Supported by our landmark observations, it has been suggested that dysregulations in the HPA axis caused by chronic pain may lead to activating pro-inflammatory mechanisms, directly impacting mental health and, potentially, lesion growth. Therefore, our well-controlled studies in the animal model, together with the clinical literature, suggest that targeting the HPA using stress relief should alleviate inflammation and alter responses to pain. These data support the unique opportunity of altering brain-body-brain pathways as potential complementary and interdisciplinary therapeutic options for endometriosis (29).

Prior research in the field of complementary and integrative medicine has shown that stress-coping strategies (yoga, tai-chi, support groups) have beneficial effects on chronic pain conditions, inflammation, immune and brain function, as well as anxiety, depression, and stress levels (63–67). Disease processes exacerbated by stress can be interrupted by psychological and stress reduction interventions, including social coping, psychotherapy, exercise, relaxation, meditation, and yoga (68–70). Because chronic pelvic pain, a hallmark of endometriosis, is associated with multiple factors (social, biological, psychological), its clinical management is challenging. Many patients remain in pain despite multiple interventions involving hormonal therapies and surgery. Despite strong evidence that women with endometriosis have a high prevalence of substantial stress levels and of a strong association between stress and disease severity (29), there has been limited investigation regarding integrative medicine alternatives for endometriosis, with few published RCTs (46, 59, 71–73). As a result, patients are still at a loss concerning alternative options for managing symptoms as well as their impact on emotional wellbeing and mental health when conventional therapies fail or provide only partial, short-term relief.

To provide patients with alternative non-pharmacological, non-surgical adjuvant treatment approaches for managing endometriosis-associated pain and mental health disturbances, our team used a mixed methods approach for translating the EE paradigm into a pilot integrative multi-modal intervention. We showed that the intervention was *feasible* based on the total number of patients who met the inclusion criteria and were randomized. The enrollment rate was low due to the various challenges we had to tackle during the study. First, we had to postpone the start of the intervention due to earthquakes (January-February 2020) and then the COVID-19 lockdowns and restrictions (March 2020-March 2021). After a one-year hiatus, the study started in August of 2021 when the restrictions for gatherings were suspended, and vaccination and treatments were available. However, this delay meant that we had to re-start our recruitment campaign for the first intervention session. Of note, both EE sessions were conducted during the COVID-19 pandemic (August 2021-February 2022), which must be considered when analyzing the results. However, none of the subjects identified the pandemic as a reason for not participating or missing an intervention.

Adherence rate was lower for the intervention group than the control group. This was expected as the intervention involved traveling to different venues and a commitment of four hrs on six alternating Saturday mornings, while controls were visited in their homes or meeting points for sample and survey collection and had a minimal time commitment (∼30 min). The most common reason for missing an intervention was pelvic pain, which in patients with endometriosis is unpredictable and incapacitating, followed by work/study issues. Though some patients needed to travel to distant locations (30 min–1 h) during the study, most enrolled participants did not consider this an inconvenience. However, this was a factor impeding participation for some of the screened potential participants. Future interventions should consider the distance to venues to overcome participation limitations due to transportation issues. There was a dropout rate of 30% in the control group which can be ascribed to the natural emergencies experienced during the study. Participation rates were 58.6% for at least a half dose (≥3 interventions or more) and 41.4% for the full dose. In future interventions, make-up sessions could be offered to solve attendance issues due to pain or work/family conflicts.

Despite the low enrollment and adherence rates observed in the intervention group, the intervention was highly acceptable (>70%–96% positive evaluation of the logistics) and perceived as valuable by participants, according to both quantitative data (post-intervention evaluation and qualitative data at 3-month follow-up focus group). Survey completion rates were very high (>80%) throughout the intervention timeframe but dropped substantially 3-months post-intervention. The support group component of the intervention was perceived as Excellent by 91%, and 82.4% said they would participate regularly. Most participants (96%) said they would recommend the intervention to other patients. Although there are other available support systems for women with endometriosis such as online groups and chats, the structured nature of the EE intervention may have played a role in the high acceptability rates shown by this RCT.

To our knowledge, translation of the EE paradigm to clinical use in pain/inflammatory disorders has not been undertaken, despite ample evidence from animal models alluding to its anti-stress and anti-inflammatory effects. Others have commented on the challenges of translating this multi-modal systematic approach consisting of psychological (group support), behavioral (novelty), and stress-reduction (open environments) to the human scenario (74). In our experience, the major challenges to implementing the EE intervention included unpredictable events that impacted enrollment (e.g., the COVID-19 pandemic) and conflicts due to work/study that impaired participation. Effective delivery of the EE program would require significant human resources, which we solved by leveraging advanced graduate (MD, PsyD, MPH, nursing) students in our institution. An MoP with detailed step-by-step protocols and videos will be available to psychology professionals to enable the intervention to be replicated similarly, regardless of location. This pilot RCT demonstrated high acceptability of patients of this multi-modal integrative medicine approach and uncovered challenges and opportunities for improvement to ensure feasibility. Additional studies in a diverse patient population are warranted to ensure the generalizability of its findings. Future analysis of the efficacy data collected during this study will assess whether this intervention produces significant positive outcomes in endometriosis patients regarding pain and stress perception, mental health, and QoL.

## Data Availability

The raw data supporting the conclusions of this article will be made available by the authors, without undue reservation.
